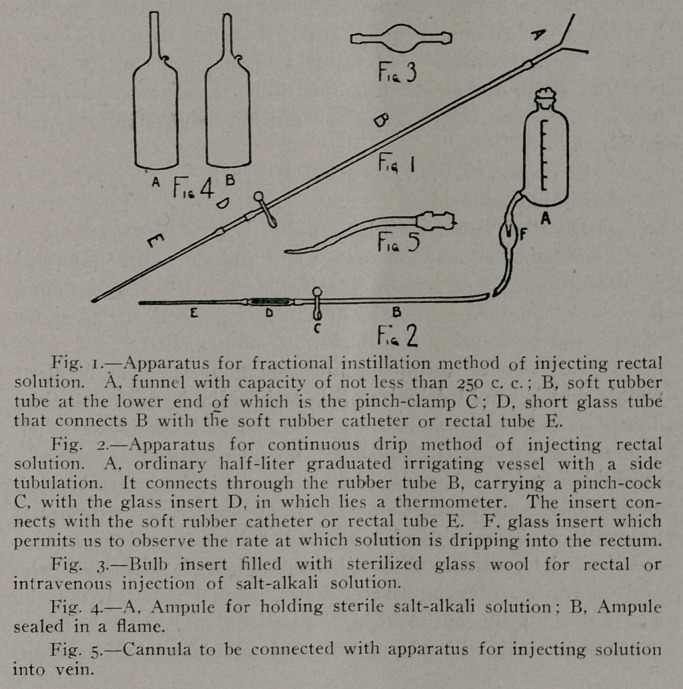# Selections and Abstracts

**Published:** 1913-06

**Authors:** 


### SELECTIONS AND ABSTRACTS

### WHY SHOULD BIRTHS AND DEATHS BE REGISTERED?

Proper registration of births and deaths is of great importance to the adult members of any community. Not only are such records necessary for the accurate study of disease and its prevention, but they are also of the utmost importance in all questions relating to heredity, legitimacy, property rights and identity. No child labor law is of value, unless it rests on a system of birth registration and birth certificates, by which the child and the parent can be required at any time to produce positive proof of the age of the child. Laws regulating the age of consent cannot be rigidly enforced, so long as the question of the age of the girl depends on the statements of interested persons rather than on official state records. In practically all other civilized nations, proper registration of births is accepted as a matter of course. Europeans look with astonishment upon the American people, when they learn that there are at present only eight American states which have any adequate birth registration. These are: Maine, Vermont, New Hampshire, Massachusetts, Connecticut, Rhode Island, Pennsylvania and Michigan. Resolutions adopted by the General Federation of Womans Clubs at San Francisco recognized the fact that proper registration of births is absolutely essential for the effective operation of the Children's Bureau recently established by the Federal Government of which Miss Julia Lathrop, of Chicago, has been made the Director.

Equally important is the proper registration of deaths. No civilized community should allow a human being to die and be buried without a proper official record having been made of the fact. Such records are indispensable in determining death-rate, proportion of deaths and births, duration of life, rates of life insurance, etc., and in preventing and detecting crime.

In preparing legislative bills on most subjects, the first question for consideration is the drafting of the bill. In this case, fortunately, it is not necessary, as a model bill on this subject has been in existence for some time. The text of this bill appears on page 13 of this pamphlet. It was drafted in 1907, patterned after the Pennsylvania law, which has proved most effective.

This bill has since been endorsed by the Census Department of the United States Government, the American Medical Association, the American Public Health Association, the American Statistical Association, the Committee on Uniform Laws of the American Bar Association, the American Association for the Study and Prevention of Infant Mortality, the general officers of the American Federation of Labor and the National Conservation Congress, as well as by numerous local and state associations and other public health bodies. It can, therefore, be said to represent the combined judgment of all those interested in securing better vital statistics, legislation and registration for the United States, as well as the knowledge and experience of those best qualified to speak with authority on the subject.

It is especially recommended that every effort be made to secure the cooperation of civic federations, women's clubs, public health leagues, labor organizations, local boards of trade and business men's associations and other similar bodies. In spite of the fact that no personal benefit accrues to any physician from a law requiring registration of births and deaths, and that most of the labor falls on the physician, the activity and prominence of the medical profession in securing the passage of such laws has created the impression in the public mind that these measures are in some way of personal benefit to physicians and are of no particular interest to the general public. That this is not the case will be seen by considering the importance of vital statistics legislation to the public and its relation to all the important events of life. Public interest in his question should not be allowed to subside until adequate laws have been passed in every state requiring the proper registration of births and deaths and making at least as lasting a record of the most important events of individual existence as is made of blooded horses, cows, dogs and cats. The neglect of such matters shown by our American states is a source of never-ending wonder to European visitors and reflects no credit on our civilization. —(U. S. Department Commerce, Bureau of the Census.)

---

### THE TREATMENT OF AMEBIC DYSENTERY WITH SUBCUTANEOUS INJECTIONS OF EMETINE HYDROCHLORID.

Randolph Lyons, M. D., New Orleans.
Instructor in Clinical Medicine, Tulane University

(Continued From Last Issue.)

December 4: Bowels have acted once or twice daily since last injection but abdominal discomfort (described by patient as "boiling" sensation) still persists. Stool contains some mucus but no amebas. Both arms sore and infiltrated around sites of injections. Emetine hydrochlorid ⅝ grain by needle.

December 12: Some abdominal pain still present. Patient had three soft stools, but the last one showed a few streaks of bloody mucous; active amebas found; no trichomonads.

As the previous emetine solution was so irritating it was discarded for a fresh one. Emetine hydrochlorid ⅝ grain by needle.

December 13: No stool. Emetine hydrochlorid ⅝ grain by needle.

December 14: One action: contains no mucus, blood or ameba. Patient feels much better.

December 20: The patient was given three injections of 2-3 grain a day of preparation of emetine hydrochlorid put up in ampules on the 17th, 18th, and 19th, in order to make assurance doubly sure.

December 27: Bowels normal for two weeks. The injections caused practically no pain or tenderness. Patient discharged.

The failure of the first four injections may perhaps be attributed to the local infiltration caused by that particular preparation with consequent delayed absorption.

Case 6.—White man, aged 40, cashier, home, New Orleans, was seen Dec. 5, 1912. Present illness began about three weeks ago with loose bowels. Stools contained mucus. He was treated by a colleague at office with small doses of Epsom salts and enemas. As bowels did not improve at the end of a week, he was put to bed. Stools now contained bloody mucus. On several occasions he passed over one-half ounce of pure blood. As his physician was called out of town, I was called in to see him. He complained of no pain. Temperature was normal. Stool consisted chiefly of blood-streaked mucus.

Physical Examination.—Patient well-nourished. Passages number about ten in twenty-four hours. States he has lost 10 pounds. Thorax, negative. Abdomen, negative.

Course and Treatment.—December 6: Stool (obtained by rectal tube) shows mucus, blood, pus and a few motile amebas, no trichomonads.

December 7: Emetine hydrochlorid ⅝ grain by needle.

December 8: Three stools since last injection, last one partially formed. Emetine hydrochlorid ¾ grain by needle.

December 9: Three stools, soft, a little mucus still present. Emetine hydrochlorid ⅝ grain by needle.

December 10: One stool since injection; no blood, pus or amebas. Both arms slightly painful and tender.

December 14: Bowels constipated. Given purgative, full diet. Discharged.

December 16: (Office) Patient weighs 167 pounds, usual weight 187 pounds.

December 21: (Office) Bowels regular and normal.

March 7, 1913: Back to former weight. Bowels normal.

Of the six patients all recovered except Patient 2. In fairness, however, this case should be omitted in judging the treatment, as the patient was beyond redemption. The average length of treatment with emetine until stools became normal was nine days for the five cases. The shortest interval was two days and the longest twenty days. There is little doubt but that this average of nine days would have been considerably lower had the ampules of the emetine been used throughout. For example in Case 3 injections had to be discontinued temporarily because of the pain produced. In case 5 absorption was most probably delayed because of local infiltration. The largest dose used was ¾ grains. Larger doses may be found more effective. There were no ill effects noted from this dosage. The average total amount of emetine hydrochlorid used for the five cases was 2.6 grains. This is 0.6 of a grain more than Rogers required for cure in his series. In a case under observation at present 2 grain of emetine have been given and stools have been normal for the past three days.

### Advantages of Emetine Treatment

The treatment of amebic disease with emetine rests on an experimental basis. It has been shown that ipecacuanha without emetine has but little effect on ameba in vitro (as well as clinically) while emetine has a most powerful amebicidal action. Granting this, then we may assume that emetine is the active principle of ipecac so far as amebas are concerned and theoretically should be used in preference to the whole drug in the same manner as we employ quinin for the treatment of malaria in preference to cinchona. According to Rogers a third of a grain of emetine is equivalent to 30 grains of ipecac. The soluble salts of emetine are put up by several pharmaceutical houses. The hydrochlorid, to which my experience is limited, is practically nonirritating when used subcutaneously and in moderate doses causes no nausea, vomiting or depression. Rogers' largest dose was 3 grains in one day. Allen injected 4 grains at one dose and produced nausea for several hours—the patient vomiting once. Rogers has given the hydrochlorid intravenously (one grain in 5 c.c. of normal saline solution) without any depressing effect on the pulse.

To summarize, the advantages of the emetine treatment are briefly (1) simplicity and ease of administration of the drug; (2) no vomiting or depression; (3) accurate dosage (no loss through bowels); (4) rapid absorption and effect; (5) reliability of product (hydrochlorid).

### Does it Cure?

The question of curability of the emetine treatment can be answered only after a long period has elapsed. Of the five patients that recovered, four have been heard from or seen and all have remained well. Patient 1 is still cured after three months; Patient 4 after three months; Patient 6 after two months and three weeks. Patient 5 has unfortunately been lost, the outlook is, however, very encouraging.

While no definite conclusions can be drawn from the observations of so small a number of cases, I believe that the results are highly suggestive that in the subcutaneous injections of soluble emetine salts an ideal method has been found of treating amebic disease. Time will soon show whether or not, as Rogers believes, another specific has been found.—Jour. A. M. A., April 19, 1913.

### THE TREATMENT OF NEPHRITIS AND ALLIED CONDITIONS.*

Martin H. Fischer, M. D., Cincinnati.

It is now almost two years since the first attempt to utilize the following views was made by James J. Hogan, who relieved a case of acute nephritis with anuria by injecting an alkaline hypertonic sodium chlorid solution. Since that time the simple principles of treatment proposed have been tried on many private and hospital patients, so that it has been possible to arrive at some conclusions regarding their actual value. I wish to discuss this question briefly in the following pages. At the same time, in order to satisfy a number of medical men, I desire to outline in great detail the method of treatment which will in my judgment insure the best results.

For a detailed statement of my colloid-chemical views on the nature and cause of nephritis I must refer the reader to previous discussions. (1.) They may be briefly expressed as follows: All the signs and symptoms of nephritis are colloid-chemical in nature, and in the main are the result of a common cause, and abnormal production or accumulation of acid in the kidney. The action of the acid in making the protein colloids of the kidney go into solution in the urine explains the albuminuria; its action in making the kidney-colloids swell causes the increase in the size of the kidney; its action in precipitating a second colloid makes the change of color (graying) in the kidney. Since the (colloidal) cement-substance that holds the kidney-parenchyma fast to its supporting tissue dissolves more

*The relief of glaucoma through rectal injections of alkali and salt is discussed in the author's reprints.

*From the Eichberg Laboratory of Physiology in the University of Cincinnati.

1. Fischer, Martin H.: Edema, New York, 1910, p. 191; Further Remarks on a Colloid-Chemical Analysis of Nephritis, Kolloid Ztschr., 1911, viii, 202; Contributions to a Colloid-Chemical Analysis of Absorption and Secretion (Absorption from Peritoneal Cavity), Kolloid-chem. Beihefte, 1911, ii, 304 (available in English in Cincinnati Lancet-Clinic, 1912, cvii); Practical Points in the Treatment of Nephritis, Ohio State Med. Jour., Aug. 15, 1911; Nephritis (a monograph dealing specifically with this subject), New York, 1912.

easily than that which binds the cells to one another, the cells slip out of the uriniferous tubules in groups called "casts." The casts may be cellular, granular or hyaline, depending solely on the state of the colloids found in them. This state in its turn is determined by the amount of acid, salt, etc., present in the blood, the kidney or the urine. The changes in the output of water are referable to a disturbance in the normal dynamic relations existing between the concentration of water and acid found in the blood, and the water and acid found in the kidney-parenchyma. The absolute decrease in the amount of dissolved substances secreted is held to be secondary to the absolute decrease in the amount of water secreted by the nephritic kidney. The change in the relative concentration of the dissolved substances in a nephritic urine as compared with the relative concentration of the same substances as observed in normal urine is dependent on changes in the adsorption characteristics of the blood and kidney-colloids, as influenced by such a change in the composition of the kidney as is determined by an abnormal production or accumulation of acid in this organ.

I maintain that all nephritides are parenchymatous. These may be general (involve the whole kidney), as is the case in the ordinary acute or chronic intoxications; or focal (involve only parts of the kidney), as is the case in the so-called chronic interstitial nephritides associated with cardiovascular disease (primary contracted kidneys). In the latter, in consequence of changes in the blood-vessels, one fragment after another of the kidney is destroyed and replaced by connective tissue; but between these spots the kidney substance is largely normal, and so the decrease in urinary output, the albuminuria, the casts, etc., may be largely absent.

I wish to direct attention to two more points in order to render more clear the purpose of my therapy. It is universally held that the generalized edema of a patient is secondary to the kidney disease. This is, in the main, incorrect; for nephrectomized animals either develop no edema at all or only a very slight one as compared with the edema developed, for instance, after the injection of uranium nitrate. I believe that the generalized edema and the edema of the kidney (nephritis) arise simultaneously and from the same cause.

The other point is that there is no free water in the body; it exists in all the tissue- and body-fluids only in combination (as water of hydration) with the various (hydrophilic) colloids present. When any free water appears in the body, it is quickly removed by one of the secretory organs (such as the kidney). Conversely, it is impossible to get any secretion except as we furnish the secreting organ free water.

Text-books list as "causes" of nephritis, excessive muscular work, heart-disease, lung-disease, anemia, carbon-monoxid poisoning, exposure to cold, interference with the blood-supply to all or to a part of the kidney (pressure on the kidney-vessels, arteriosclerosis, thrombosis, embolism), intoxication of the kidney-parenchyma with a toxin, chloroform, ether, arsenic, uranium, chromium, lead, phosphorus, amyl nitrate, etc., restriction of salt consumption or excessive consumption of water low in salts. These all gain their etiologic importance because they represent methods which directly or indirectly make for an abnormal production or accumulation of acid in the kidney.

These remarks will suffice to indicate why I have formulated the general rule for the prophylaxis and treatment of nephritis in the following terms: As far as possible, avoid and combat every condition that favors the abnormal production or accumulation of acid in the kidney. Evidently the pathologic condition of the patient must be taken into consideration in the aplpication of this rule. Naturally an anesthesia nephritis with suppression of urine will call for a more aggressive therapy than nephritis secondary to a slowly progressing arteriosclerosis. If we succeed in getting the first nephritic over his immediate kidney symptoms, we may make a hopeful prognosis, for when he has exhaled his anesthetic he has rid himself of the condition that was responsible for the abnormal acid content in his kidneys. But in the case of the second nephritic so hopeful a prognosis cannot be made, for while we may also benefit him, he continues to carry the original condition that brought him to us—his arteriosclerosis—even after we have treated him.

My rule for the treatment of the threatened or established case of nephritis may be summarized in these words: Give alkali, salts and water. The reasons in brief are as follows: The alkali is given in order to neutralize the acid present in abnormal amount in the kidney and in the other edematous organs of the body. The salts are indicated and sodium chlorid is no exception) because the various changes induced by the kidney-colloids by acids are counteracted by adding to such acid any salt, even a neutral salt. (2.) We need to give water in order to have more of this present in the body than is necessary to saturate all the body-colloids; otherwise we shall have no "free" water left for the secretion of urine.

### The Prophylaxis and the Treatment of the Milder Cases of Nephritis.

First of all, it is necessary to get as clear a conception as possible of all the factors that conspire toward the production or the maintenance of an abnormal acid content in any case of nephritis. (3.) Usually there is more than one factor. To the toxic cause of a scarlatinal nephritis may be added that of inadequate oxidation if the patient develops bronchopneumonia. To the toxic nephritis of a pneumococcus infection, already aggravated by interference with respiration, may be added an additional item if the patient develops a convulsion, and so (through muscular work) produces suddenly an enormous additional amount of acid. The eclamptic who has just managed to drag through the last weeks of pregnancy faces death when the muscular efforts of labor or of a convulsion add more acid to that resulting from the intoxication of pregnancy. The patient with subacute or chronic nephritis, who, with persistent edema,

2. Fischer, Martin H., and Moore, Gertrude: The Swelling of Fibrin, Am. Jour. Physiol., 1907, xx, 330; Kolloid Ztschr., 1908, v, 197. Fischer, M. H.: Further Experiments on the Swelling of Fibrin, Arch. f. d. ges. Physiol. (Pfluger's), 1908, cxxv, 99; and subsequent papers.

3. The term "nephritis" is used throughout this paper in its ordinary clinical sense as covering that symptom-complex which is characterized by the appearance of albumin and casts in the urine, by changes in the quantitative output of water and dissolved substances by the kidney, by the development of edema, etc.

some casts, albumin, and a deficient output of urine, is permitted to walk about the ward, may be keeping up the acid content of his tissues by even such light muscular work.

After we have removed as many of these conditions as possible, we may turn our attention to combating those which we cannot remove. These we must try to render as innocuous as possible. Directly or indirectly, we shall find ourselves face to face here with the necessity of fighting an intoxication. In an infectious disease we wish to keep the concentration of the unknown toxin produced as low as possible. The toxin is responsible for the abnormal production of acid in the cells of the kidney (and other organs), and I hold this to be the direct cause of the nephritis. But it should be recalled that the acid intoxication is itself proportional to the concentration of the acid, and this too we wish to keep as low as possible. This can be done only by giving water. It must be insisted on, moreover, that the administration of water shall be regular, and not left to the haphazard inclination of the patient. A good rule is to give a glass every hour day and night. The night administration of water is as important as that through the day, for the production of toxin does not stop with nightfall.

If the water contains an alkali of some sort, so much the better. The natural or artificial alkaline spring-waters are perhaps most easily borne. If the patient will tolerate it, 0.5 to 1.0 gm. of sodium carbonate or sodium bicarbonate may be added to each glass of such alkaline water or to plain water. Some patients who cannot tolerate the carbonates will take sodium citrate, sodium tartrate, sodium acetate or other salts of a strong base with a weak acid in half-gram, or larger doses every hour, either when dissolved in water or in capsules followed by water. Calcium hydroxid can easily be given by mixing limewater with milk. As the salts of the bivalent metals are particularly active in decreasing the hydration-capacity of the body-colloids, the administration of magnesium oxid or milk of magnesia up to the point where two or three easy movements of the bowels are obtained daily, give good results. The soluble salts of calcium and strontium, as well as their iodids, acetates, lactates, citrates or tartrates, are also available.

The dietary can be made and excellent and natural vehicle for getting alkali and salts into the body. A predominantly vegetable diet means a diet rich in alkali. Instead of restricting the protein intake of the patient as greatly as has been the custom, it is better to allow a moderate protein ration on the condition that the patient will greatly increase his consumption of selected fruits and vegetables. It is well to bear in mind that vegetables and fruits (with the exception of those that are high in oxalic acid) will yield a greater proportion of alkaline and neutral salts when cooked than when raw. The sweet fruits, either raw or cooked, constitute a most agreeable form in which to get an excess of alkali into a patient.

For reasons that I have often discussed, I think that the amount of table-salt should not be restricted, but that, on the contrary, it shoudl be urged on the patient. Food serves as a natural carrier for this. In the form of salt meats and salt fish we can easily get considerable quantities of sodium chlorid into our patient, and if he keeps a salt-shaker at hand he can liberally increase his intake by dusting salt on his vegetables, his fats and such proteins as we allow him. Through the diet alone we can therefore do a great deal to keep the intake of alkali, salts and water high.

Let us now imagine that, in spite of these procedures, the symptoms of the nephritic do not improve or that the symptoms are so severe from the start that alkali by mouth seems inadequate. What are we to do then?

We continue to utilize the gastric route as well as we can, but now make use of the rectum also, in order to get a further absorption of alkali, salt and water. We never seriously consider whether the patient ordinarily drinks distilled water, or tap-water, or a table-water, and from this point of view we might be inclined to ignore the exact composition of the liquid to be injected into his rectum. But it should be remembered that the proper regulation of salt-consumption is accomplished through the "taste" of the individual. If his salt-consumption has been too high, he craves fresh water and so washes out the excess of salts. If, on the other hand, he has lost too great an amount of salt from his tissues, he consumes more salt and makes up the deficit. So when we give a nephritic water by rectum we must try to give him at least enough salt to cover also the washing-out effects that would follow if we gave pure water. Therefore the substitution of a "physiologic" sodium chlorid solution for water, or better still, a "Ringer" solution, is eminently in order.

But a nephritis has other needs than a normal individual. His kidney specifically, and all the rest of his organs generally, are suffering from an abnormal acid content; that is, they have an increased capacity for holding water (an increased tendency to swell). To reduce the edema of the kidney or in the brain, in the optic nerve or in the tissues generally in the nephritic, I therefore try to increase, at least temporarily, the absolute concentration of salt in the whole body. This is best accomplished by using a hypertonic salt solution (4) to which is added an alkali. Obviously when using such a hypertonic solution by rectum (or intravenously) I do not allow any water to be taken by mouth. The patient may wet his mouth to relieve his sense of thirst, but no more; otherwise we should only be reducing the concentration of the solution we are administering:

The following formula works very well:

Sodium carbonate (Na2CO3.10 H2O) . . 10 gm
Sodium chlorid . . . . . . . . . . . . . . . . . . . . 14 gm
Distilled water, enough to make . . 1000 c. c.

Simple as this formula is, care must be taken in its preparation if good effects are to be expected. Sodium carbonate is

4. The osmotic conception of water absorption has assumed such dominance in the biologic sciences that the word "isotonic" has been made synonymous with "isosmotic," while the words "hypertonic" and "hypotonic" have come to mean respectively, osmotic concentrations above and below this. As we can no longer uphold the osmotic theory of water absorption by protoplasm, we must again learn to use the words "isotonic," "hypertonic" and "hypotonic" in their original sense, "isotonic" referring to solutions of any substance or substances which allow of no change, "hypertonic" to those permitting a decrease, and "hypotonic" an increase in the volume of the cell, tissue or organ.

used, not bicarbonate. The carbonate is physiologically more effective than the bicarbonate, for one of the acids which are produced in the body is carbonic acid, and sodium bicarbonate is already saturated with this, and in consequence cannot act as a carrier. The chemically pure, crystallized sodium carbonate ($Na_2CO_3 \cdot 10\,H_2O$) or the monohydrated form ($Na_2CO_3 \cdot H_2O$) is to be insisted on. Two other forms of sodium carbonate are found in the market, the dry ($Na_2CO_3$) and the so-called dry or "dried." The "dried" salt found on the ordinary drug-shelf contains approximately two molecules of water of crystallization ($Na_2CO_3 \cdot 2\,H_2O$). I am inclined to advise against all except the large crystallized form or the monohydrated form, but WHATEVER SALT IS USED, ITS WATER-OF-CRYSTALLIZATION CONTENT MUST BE REMEMBERED, OTHERWISE A SOLUTION OF A DIFFERENT STRENGTH FROM THAT WHICH I HAVE FOUND MOST USEFUL WILL BE OBTAINED. The proportionate amounts of these four salts that may be used are to each other as their molecular weights, or in definite terms:

10 gm. $Na_2CO_3 \cdot 10\,H_2O$ (mol. wt. 286, crystallized "salsoda")=4.95 gm. $Na_2CO_3 \cdot 2\,H_2O$ (mol. wt. 142, "dried" "salsoda")= 4.33 gm. $Na_2CO_3 \cdot H_2O$ (mol. wt. 124, monohydrated "salsoda")=3.71 gm. $Na_2CO_3$ (mol. wt. 106, really dry "salsoda").

The sodium-chlorid-sodium-carbonate solution should be made up in DISTILLED water and filtered. If the salts used are pure and the whole is properly prepared, the resulting solution is perfectly clear.

RECTAL INJECTION OF THE SOLUTION. Unless the patient is mentally incapable of comprehending what we say, it is well to explain to him before an injection is made just what we desire to accomplish and to secure his cooperation. As the solution is hypertonic and contains alkali in addition, it not only irritates the rectum somewhat, but leads temporarily to a secretion of water into the rectum while the salt and alkali are being absorbed. (5.) If the patient is made comfortable in bed and if his cooperation is secured, the solution is retained for longer periods of time, or entirely, and a more perfect absorption of the alkali and salt is obtained.

To inject the solution, I make use either of a continuous drip-method, or inject larger quantities at varying intervals of time. It is not necessary first to cleanse the rectum locally, and especially are we NOT to try to accomplish this end by a previous administration of cathartics. Both of these methods only increase the irritability of the rectum.

THE FRACTIONAL INSTILLATION METHOD.—This is easily accomplished by utilizing the apparatus shown in Figure 1. The solution to be injected is heated to 105 F., and the patient being in position, the catheter is lubricated with petrolatum. Into the funnel are now poured 250 c. c. of the solution, and the stop-cock is temporarily opened so as to drive the air out of the tube and catheter. The catheter is then gently inserted well into the rectum, and the funnel is emptied by again opening the pinch-cock. The short glass tube will inform the operator when the last portions of the mixture are flowing into the rectum, when the hold on the pinch-cock is released. In this way no air will be allowed to enter the rectum and balloon it, and extra irritation from this source will be avoided. The injections may be repeated as often as the symptoms of the patient demand them, or until the patient finds it difficult or impossible to retain them. A period of rest should then be given. If the case is so urgent that this cannot be allowed with safety, then the solution must be given intravenously. (See below.)

CONTINUOUS DRIP METHOD.—When the solution is to be given by this method, the arrangement shown in Figure 2 works very well. From 1 to 4 drops a second should enter the rectum. This is about as high a rate of injection as the patient

---

5. No solution is absorbed or secreted "as such," as is usually taught. See Fischer, M. H.: Edema, New York, 1910, p. 200; Kolloid-chem. Beihefte, 1911, ii, 304 (see Note 1) (available in English in Cincinnati Lancet-Clinic, 1912, cvii); Nephritis, New York, 1912, p. 113; Hogan J. J., and Fischer, M. H.: The Theory and Practice of Perfusion, Kolloid-chem. Beihefte, 1912, iii, 385.

can stand without rejecting the fluid. Roughly, this corresponds to an injection of from 240 c. c. to 960 c. c. per hour.

The injection-fluid is retained best if it is delivered INTO THE PATIENT at not less than body temperature, and 110 F. is better. For this reason the thermometer in D, located as near the rectum as possible, is of great convenience. As the solution slowly passes out of A through the tube, it falls in temperature. The vessel A is therefore conveniently filled with the solution at a temperature somewhat above 110 F. Or one can set this vessel into a second one containing warm water, or place the tube B in warm water, or cover it with a blanket by way of maintaining the solution at a proper temperature, as convenience and the ingenuity of the medical man dictate. In hospital practice, thermostatic devices heated by electricity or gas may be installed conveniently.

AMOUNT AND TIME-INTERVAL.—How much of the solution may be given by rectum and how long do we continue with it? The answer to this is found in the condition of the patient. So far as I have been able to observe, no harm can be done by indefinite use of the solution. A full physiologic effect is obtained when the patient is kept free from the various signs and symptoms of nephritis, and the urine is persistently neutral in reaction toward litmus. Such a full effect may be obtained in a day or two, or it may require a week. The symptoms may have cleared entirely even before the saturation of the body with alkali has been carried to the point where the patient secretes a persistently neutral urine. His improvement will by itself suggest a reduction in the number of injections or their entire abandonment.

Individuals differ greatly in their behavior toward these injections. I have seen patients bear them for weeks at a time without complaining and without ever rejecting them. Others will insist from the first that they cannot hold them. In this connection, it is well to point out that if these hypertonic salt-alkali mixtures are retained for any time at all, say even for an hour, they do much good. When at the end of such a period the patient rejects some fluid from the bowel, this is not the same as that which was introduced, simply minus a certain quantity that has been absorbed. Hypertonic sodium chlorid-sodium carbonate mixtures are not absorbed as such. The salt and alkali are absorbed out of the solutions while water is being secreted into the bowel. If the solution is retained even for a short time, the patient will have increased his body-content of alkali and salt, which is the whole purpose of my therapy.

It is well, if possible, in the first twenty-four hours of treatment to have 2 liters (about 2 quarts) of the solution retained by the patient. For a few hours after starting the injection it is well to give but little water by mouth, in order to exert as great a shrinking effect as possible on all the tissues.

But as soon as the urinary output begins to increase and rise toward a more normal point, I urge the patient to drink water, so as to have free water available for urine. I find good practitioners overlooking the importance of this point constantly. While some water is introduced with the hypertonic salt solution, most of the "free water" that is lost through the kidney after absorption of the hypertonic solution has been taken from the tissues (which may be edematous). But it is self-evident that this robbing of the tissues can go on only for a limited time. Then we have to supply water from the outside if we wish to keep up the urinary secretion.

If for any reason it is not possible to feed enough water by mouth, then we can use the rectum. After the kidneys are functioning in a more normal way, we may substitute for the hypertonic solution one more nearly isotonic with the body-fluids. A "physiologic" salt solution (0.85 per cent. to 0.90 per cent. sodium chlorid) does very well, though the following mixture is better, for it gives the much needed alkali as well as the water.

| | |
|---|---|
| Sodium carbonate ($Na2CO3.10\ H2O$) . . | 10 gm. |
| Sodium chlorid . . . . . . . . . . . . . . . . . . . . . | 7 gm. |
| Distilled water enough to make . . . . . . | 100 c. c. |

This solution may be injected in indefinite amounts and gives rise to no rectal irritation. But since it is not hypertonic, it does not produce so marked or so rapid general effects as we seek in the more acute manifestations of nephritis.

The desire to introduce into the nephritic the bivalent metals which would dehydrate his body-colloids far more than do the univalent metals that are used cannot be easily satisfied. The reason for this is obvious. Their hydroxids are largely insoluble, and so the administration of bivalent metals such as calcium or magnesium along with carbonates or hydroxids is impossible. The only schemes that I have found of service are limited to patients with mild nephritis and to patients who are recovering from the severer types, when lime-water may be added to the milk consumed by the patient; or he may be given magnesium oxid, milk of magnesia and soluble calcium or strontium salts by mouth. For rectal injection a "physiologic," 0.85 per cent., sodium chlorid solution to which not more than 0.1 per cent. calcium chlorid is added also works very well if alkaline solutions have not been used for some hours previously.

### The Treatment of Severe Cases of Nephritis.

Under this heading I shall consider those patients in whom we encounter such especially alarming signs and symptoms as great or complete suppression of urine, rapidly progressing optic nerve changes, persistent headache and nausea, vomiting, convulsions, stupor and coma, great quantities of albumin in the urine, etc.

While I realize that a complete explanation of the nature and of the cause of these various clinical signs cannot be summed up in any brief statement, I believe that an essential, if not the essential element in all of them is an edema of the affected part. This edema is represented physico-chemically by an increased swelling on the part of the colloids of the tissues involved, and as responsible for such an increased swelling we hold the abnormal production or accumulation of acid in the part, either alone or in conjunction with such other substances as are also capable of increasing the hydration-capacity of the tissue-colloids. Or, to repeat what I have already often emphasized, what are called the serious complications of nephritis are not really complications secondary to this pathologic entity, but are maifestations in other organs of the body of the thing which in the kidney we call nephritis. The lesions in other organs are as primary as those in the kidney.

Just as the nephritis is in large measure an edema of the kidney, so the optic nerve swelling and the "retinitis" of nephritis are edema of the optic nerve and of the retitna; the headache, convulsions and coma are manifestations of an edema of the brain; the persistent nausea and vomiting of central origin are manifestations of an edema of the medulla; and the generalized edema is an expression in the body-tissues of that which in the kidney we call nephritis. The same intoxication underlies all these changes, and it is mere accident, not immediately analyzable, that one nephritic will show particularly prominent eye-symptoms, another a generalized edema, while a third will call us to his side because of a convulsion. For any one of a number of reasons, the edema may become particularly prominent in his optic apparatus, or in his body-tissues generally, or in his brain. If these facts are borne in mind, it will serve to indicate why I believe that any or all of these conditions demand the same general treatment, and why, if I succeed in combating a particularly prominent one, I find that I have succeeded in combating all the rest as well.

Whether the particularly alarming symptoms arising from the kidney itself (a suppression of urine, a great albuminuria, etc.), or whether they spring from the brain (convulsions, stupor, coma), the eye ("papillitis," "retinitis," partial blindness), or the medulla (nausea and vomiting), the purpose of my therapy is the same—I wish to stop and reduce the swelling of the involved tissues. Naturally the best and quickest way to do this is to inject something into the blood; what this something must be I have already discussed.

The Preparation and Sterilization of the Salt-Alkali Solution.—The first formula already recommended does very well. It is well to have this solution ready for immediate use, for its preparation in sterile form takes time, and the need for it when it arises is urgent. What I have already said regarding its chemical ingredients holds here also. The finished solutions as injected into the patient must be perfectly clear and sterile. Simple as it would seem to obtain this result, it is not always obtained even when its preparation is left to trained helpers. Therefore I may be pardoned for detailing the following rules for its preparation in containers that make it available for immediate use.

Trouble arises from the fact that alkaline solutions cannot long be kept in contact with ordinary glass containers without reacting with the glass and so leading to a separation of insoluble silicates. Jena glass flasks resist better than other material, and so the finished solution may be sterilized and kept in these. For this purpose, it is only necessary to dissolve the sodium chlorid and the sodium carbonate in the necessary amount of freshly distilled water, and filter the solution through moistened filter-paper (in order to get no shreds into the solution) into thoroughly cleaned Jena flasks which have been rinsed in distilled water. It is convenient to have 2 liters of solution in each flask. The flasks are stoppered with gauze-wrapped cotton stoppers, and may be sterilized in the ordinary way by boiling. This scheme works well in hospitals or wherever storage-room is plentiful.

When needed for injection, this solution may be poured into any one of the properly sterilized intravenous injection apparatus that abound in the market. If the solutions show a precipitate in spite of the use of Jena glass containers, the clear solution may be decanted or the whole may be filtered through a sterilized funnel into the neck of which has been forced a little sterile glass wool. Or a sterilized glass bulb insert of the type shown in Figure 3 into which has been forced some glass wool may be used in the delivery tube of the injection apparatus. As the carbonates and hydroxids of the polyvalent metals are all insoluble, every piece of any injection-apparatus must be rinsed and sterilized only in distilled water. When ordinary tap-water is used, calcium and magnesium precipitates cloud the injection-mixture.

I have found it best to make up the sodium chlorid-sodium carbonate solution in ampules and then mix this concentrated solution with enough freshly distilled water to yield the proper injection-mixture at the time this is needed. One proceeds as follows: Any desired number of multiples of 10 gm. of sodium carbonate ($Na2CO3.10\ H2O$) and 14 gm. of sodium chlorid are dissolved in enough water to make 60 c. c. of finished solution. This solution is filtered and then sterilized by boiling. (6.) One or more ampules of the type shown in Figure

6. In my book on nephritis, and in a paper or two, I have cautioned against the use of excessive heat in sterilizing these carbonate solutions. The caution I find was scarcely necessary, for at the ordinary temperatures and pressures at which such sterilization is carried out, the carbonate is not decomposed.

4, A, and of about 60 c. c. capacity (if these are not available, bottles will do as well), are thoroughly cleansed, rinsed in distilled water and then boiled in distilled water to sterilize them. Into each of these, 60 c. c. of the concentrated sodium carbonate-sodium chlorid mixture are then filtered through a small sterilized funnel plugged with glass wool. When the ampules have been filled, they are sealed in a flame as in Figure 4, B. If bottles are used they are corked with sterile corks, and over these is fastened a sterilized paper hood to protected the necks from contamination. When an intravenous injection is to be given, an ampule is taken, its neck is picked with a file, and this and the lateral bead are cleaned with alcohol, and broken off. The contents are then poured into 940 c. c. of freshly distilled water, care being taken to mix the whole so that the specifically heavier salt solution may not simply settle to the bottom.

After many trials I have found this the best way to proceed; and as dispensing pharmacists in any community are willing to carry these ampules in stock, one can easily obtain fresh and clean solutions at all times. If a precipitate of silicates should be found in an ampule, one can readily avoid pouring this into the injection-apparatus, or one may filter the contents of the ampule through a little glass wool.

Technic of the Intravenous Injection of the Solution.—The experience of the past year has convinced me that it is best in making these injections to cut through the skin and expose the vein to be used clearly to view. The results are bad if one fails in his attempt to enter a vein with a hypodermic needle through the skin. The salt-alkali mixture produces a great destruction of the tissues if it is by accident injected into them.

A vein that is deemed sufficiently large is sought in the arm, in the leg, or if necessary, in the neck. As we may wish to make several injections it is advisable to pick for the first injections the prominent veins most distant from the heart. Unfortunately, in many of the conditions in which we wish to use the sodium chlorid-sodium carbonate solution, not much choice is allowed us, for the blood-vessels are so much contracted (toxemic shock?) that it is often impossible to find any usable vein below the bend of the elbow. Even here, surgeons have been unable to find the median basilic in such cases. This will explain to the reader why in extreme cases, such a vein as the jugular needs to be and has been used.

To expose the vein painlessly a few drops of a cocain or a novocain solution may be injected into the skin. If the patient is stuporous or in coma this is of course needless. The vein is freed from its surroundings and a ligature is tied about its distal end. A second ligature is thrown about the vein, and after a cut has been made into the vein and the cannula connected with the injecting apparatus has been inserted, this ligature is tied. Care is of course taken to have no air enter the vein.

The best type of cannula to use is shown in Figure 5. The two openings at the tip and laterally make it well-nigh impossible to shut off the infusion-stream by crowding the cannula against the wall of the vein. The tapering character of the cannula allows one to push it into even a small-calibered vein, and the corrugation holds the cannula in place when the second ligature is tied.

Sometimes it is better to use a large hypodermic needle in place of the cannula. The first ligature about the exposed vein then serves to steady the vein when the needle is pushed into it. When the hypodermic needle is used it is simply held in place until the injection is completed. As can easily be imagined, the use of the needle is especially convenient if one works with such a vein as the jugular. The disadvantages in its use arise from the fact that one is likely at any time to injure the blood-vessel if the patient moves, and from the further fact that the carbonate solution affects the coat of the vein and so tends to leak out from the needle after the injection is kept up for some time.

(To be Continued in Next Issue.)

## Figures and Tables

**Fig. 1. Fig. 2. Fig. 3. Fig. 4. Fig. 5. f1:**